# Retinal atrophy, inflammation, phagocytic and metabolic disruptions develop in the MerTK-cleavage-resistant mouse model

**DOI:** 10.3389/fnins.2024.1256522

**Published:** 2024-04-12

**Authors:** Julie Enderlin, Quentin Rieu, Salomé Réty, Elora M. Vanoni, Solène Roux, Julie Dégardin, Quénol César, Sébastien Augustin, Caroline Nous, Bishuang Cai, Valérie Fontaine, Florian Sennlaub, Emeline F. Nandrot

**Affiliations:** ^1^INSERM, CNRS, Institut de la Vision, Therapeutics Department, Sorbonne Université, Paris, France; ^2^Division of Liver Diseases, Department of Medicine, Icahn School of Medicine at Mount Sinai, New York, NY, United States

**Keywords:** MerTK, soluble receptor, retinal pigment epithelium, retinal atrophy, inflammation, defective phagocytosis, metabolic dysfunction

## Abstract

In the eye, cells from the retinal pigment epithelium (RPE) facing the neurosensory retina exert several functions that are all crucial for long-term survival of photoreceptors (PRs) and vision. Among those, RPE cells phagocytose under a circadian rhythm photoreceptor outer segment (POS) tips that are constantly subjected to light rays and oxidative attacks. The MerTK tyrosine kinase receptor is a key element of this phagocytic machinery required for POS internalization. Recently, we showed that MerTK is subjected to the cleavage of its extracellular domain to finely control its function. In addition, monocytes in retinal blood vessels can migrate inside the inner retina and differentiate into macrophages expressing MerTK, but their role in this context has not been studied yet. We thus investigated the ocular phenotype of MerTK cleavage-resistant (MerTK^CR^) mice to understand the relevance of this characteristic on retinal homeostasis at the RPE and macrophage levels. MerTK^CR^ retinae appear to develop and function normally, as observed in retinal sections, by electroretinogram recordings and optokinetic behavioral tests. Monitoring of MerTK^CR^ and control mice between the ages of 3 and 18  months showed the development of large degenerative areas in the central retina as early as 4 months when followed monthly by optical coherence tomography (OCT) plus fundus photography (FP)/autofluorescence (AF) detection but not by OCT alone. The degenerative areas were associated with AF, which seems to be due to infiltrated macrophages, as observed by OCT and histology. MerTK^CR^ RPE primary cultures phagocytosed less POS *in vitro*, while *in vivo*, the circadian rhythm of POS phagocytosis was deregulated. Mitochondrial function and energy production were reduced in freshly dissected RPE/choroid tissues at all ages, thus showing a metabolic impairment not present in macrophages. RPE anomalies were detected by electron microscopy, including phagosomes retained in the apical area and vacuoles. Altogether, this new mouse model displays a novel phenotype that could prove useful to understanding the interplay between RPE and PRs in inflammatory retinal degenerations and highlights new roles for MerTK in the regulation of the energetic metabolism and the maintenance of the immune privilege in the retina.

## 1 Introduction

The removal of apoptotic cells by professional and non-professional phagocytes is a central mechanism for the development and maintenance of numerous tissues in any living organism (Rabinovitch, 1995). Apoptotic cell phagocytosis is ensured by several membrane receptors that recognize, bind, and then engulf their target, specifically exposing phosphatidylserines (PtdSer) at their surface (Fadok et al., 1992, 2001). PtdSer recognition is performed either directly or indirectly by extracellular ligands acting as bridge molecules (Ryeom et al., 1996a; Nakano et al., 1997; Hafizi and Dahlbäck, 2006a; Naeini et al., 2020). One of the receptors crucial for target internalization in macrophagic cells is the Mer tyrosine kinase receptor (MerTK) and its cognate ligands Gas6 and Protein S (Graham et al., 1994; Nagata et al., 1996; D'Cruz et al., 2000; Nandrot et al., 2000; Feng et al., 2002; Hafizi and Dahlbäck, 2006a, 2006b; Burstyn-Cohen et al., 2012). MerTK has also been shown to be part of a negative feedback loop that helps control the number of targets that can be bound to the cell surface via integrin receptors (Wu et al., 2005; Nandrot et al., 2012). This retrocontrol mechanism seems to be related, at least in part, to the release of MerTK N-terminal outside the domain in the extracellular milieu referred to as soluble MerTK (sMerTK) (Sather et al., 2007; Law et al., 2015). This process occurs via an enzymatic cleavage at Proline 485, located just above the cell surface, by the ADAM17 metalloprotease in murine macrophages (Thorp et al., 2011).

In the eye, cells from the retinal pigment epithelium (RPE) are considered to be super phagocytes (Strauss, 2005; Hamieh and Nandrot, 2019). Indeed, RPE cells are post-mitotic and do not renew while facing an average of at least 25–30 photoreceptor outer segments (POS) per RPE cell. Photoreceptor (PR) cells capture light photons in their POS-containing photopigments, thereby initiating the phototransduction cascade that is at the root of the visual signal transmitted to the brain. Because they are subjected to constant light-induced stress and related proteins and lipids oxidation, photoreceptors continuously renew their POS to remain functional (Kevany and Palczewski, 2010). To regulate this process, RPE cells in turn eliminate the most aged tips of POS at an extent of 7–10% of their length every day during the whole lifespan (Young, 1967; Young and Bok, 1969). RPE cells are thus considered to be the busiest phagocytes in the body.

Another cardinal feature of RPE cells is the circadian rhythm of POS removal, as a daily peak of activity occurs approximately 1.5–2 h after light onset for rod photoreceptors ensuring low light and nocturnal vision (LaVail, 1976; Nandrot et al., 2004). This rhythmic activity is triggered by alphavbeta5 integrin receptors and their ligand MFG-E8 (Nandrot et al., 2004, 2007). Phagocytosis of cone photoreceptors—involved in color and precision vision—can happen rhythmically as well, but the time of maximum phagocytosis varies between animal species, either after light or night onset (Young, 1977; Anderson et al., 1978; Jonnal et al., 2010). More recently, new imaging techniques using adaptive optics and optical coherence tomography (OCT) suggest that in humans, cone phagocytosis bursts approximately 1 h after light exposure (Jonnal et al., 2010; Kocaoglu et al., 2016). Interestingly, POS extremities have been shown to expose PtdSer in a rhythmic fashion, indicating to RPE cells which portion needs to be removed (Ruggiero et al., 2012). Importantly, and in contrast to macrophages, RPE cells are in constant close contact with their POS targets. To keep photoreceptors functional and to avoid too much phagocytosis, the tight control of POS phagocytosis by RPE cells is crucial. Several receptors—also used by macrophages—have been shown to contribute to this regulation, including the alphavbeta5 integrin and its ligand MFG-E8, triggering the circadian rhythm, MerTK, required for POS internalization, and its ligands Gas6 and Protein S, as well as regulating receptors such as the CD81 tetraspanin, receptors from the scavenger family such as CD36 and more recently SR-B2/LIMP-2 previously only known for its lysosomal localization (Ryeom et al., 1996b; Nandrot et al., 2000, 2004, 2007; Finnemann and Silverstein, 2001; Chang and Finnemann, 2007; Burstyn-Cohen et al., 2012; Rieu et al., 2022). It is not excluded that other receptors might intervene as well, as is the case for macrophages.

MerTK cleavage has been shown to take place for both macrophages and RPE cells (Sather et al., 2007; Thorp et al., 2011; Law et al., 2015). We previously showed that POS phagocytosis increases the level of MerTK cleavage and that sMerTK levels *in vivo* vary depending on the time of day (Law et al., 2015). Notably, and in contrast with previous works, our data also suggested that MerTK ligands Gas6 and Protein S, known to stimulate phagocytosis in macrophages, appear to play opposite roles in RPE cells, Gas6 stimulating MerTK cleavage and inhibiting phagocytosis while Protein S decreased sMerTK release and increased POS phagocytosis (Ishimoto et al., 2000; Prasad et al., 2000; Anderson et al., 2003; Burstyn-Cohen et al., 2012; Law et al., 2015). Hence, the respective bioavailability of each ligand *in vivo* might contribute to sMerTK release (Nandrot, 2018). A transgenic model devoid of the cleavage site—the MerTK-cleavage resistant mouse (MerTK^CR^)—has been generated and displays better efferocytosis, improved inflammation and atherosclerosis resolution, enhanced systolic function, and smaller infarct sizes but higher fibrosis during hepatosteatosis (Cai et al., 2016, 2017, 2018, 2020; DeBerge et al., 2017; Rymut et al., 2020). These results show that, depending on the tissue, this cleavage mechanism downregulates MerTK activity and can thus have positive or negative consequences. In the eye, the importance of MerTK cleavage has not been explored yet. We thus set out to characterize the retinal phenotype and RPE function in MerTK^CR^ mice using visual phenotyping, histology, *in vitro* phagocytosis, and metabolic assays.

## 2 Materials and methods

### 2.1 Reagents, antibodies, and cell culture

Reagents were from Sigma unless otherwise stated. Antibodies used for the various experiments are detailed in Supplementary Table S1.

### 2.2 Animals and tissue collection

Wild-type (C57BL/6 J) and homozygous MerTK^CR^ mice (Cai et al., 2016) were housed under cyclic 12-h light/12-h dark conditions (light onset at 8.00 AM) and fed *ad libitum*. Animals were handled according to the Association for Research in Vision and Ophthalmology (ARVO) Statement for the Use of Animals in Ophthalmic and Vision Research and protocols approved by the Charles Darwin Animal Experimentation Ethics Committee from Sorbonne Université and the French Ministry for Education, Higher Studies and Research (APAFIS#1631–2015090415466433, APAFIS#20191–2019040311402311).

Animals were euthanized using CO_2_ asphyxiation either at 10.00 AM (time of the phagocytic peak; visual phenotyping cohorts and electron microscopy) or at different times of the light:dark cycle (analysis of the *in vivo* rhythm of phagocytosis) depending on the experiment. At the time of euthanasia, mouse ages were as follows: 3 months for the retinal structure (section 3.3) and 18 months for the retinal phenotype cohort, except ERGs collected at each age (section 3.5) or as indicated in the other sections. Complete eyes were enucleated gently using scissors to avoid pulling the retina and fixed immediately in 1 mL Davidson fixative (95% ethanol, 100% formalin [saturated aqueous solution of formaldehyde gas, 37–39%], glacial acetic acid in double distilled H_2_O) for 30 min–1 h at 4°C. After making a small opening in the cornea to let the fixative enter, eyecups were further fixed in 1 mL Davidson fixative for 3 h at 4°C. The cornea was then dissected and the lens removed while leaving the iris intact, and eyecups were fixed for 3 more hours at +4°C. Samples were further processed overnight on the Spin Tissue Processor STP 120 (Myr, Thermo Scientific), successively with dehydration and paraffin soaking steps. Eyecups were then paraffin-embedded in molds with the eye oriented, and 5-μm sections were cut.

### 2.3 Immunohistochemistry, H&E Staining, and microscopy

We usually use sections in the optic nerve area for consistency between samples. Paraffin was removed using two 15-min baths of Safesolv (Q-Path), and slides were rehydrated in gradual ethanol baths.

For immunohistochemistry labelings, antibody sites were unmasked in 1X citrate buffer heated at 95°C, and slides were allowed to cool down in the buffer. RPE pigments and endogenous peroxidases were removed in 5% H_2_O_2_, 1X SSC in deionized formamide under bright light for 15 min. Sections were permeabilized in 0.3% Triton X-100, 1X TBS, and non-specific signals were blocked in 4% BSA in 1X TBS containing 10% donkey serum. Sections were then incubated with primary antibodies overnight at 4°C (Supplementary Table S1). After three washes in 4% BSA in 1X TBS, sections were incubated with appropriate secondary antibodies (AlexaFluor, Invitrogen) for 1 h at room temperature. Cell nuclei were labeled with 1 μg/mL DAPI for 15 min at room temperature, two more washes were made, and slides were mounted using Vectashield (Vector Laboratories). For autofluorescence analysis, only the nuclei were labeled and no primary or secondary antibodies were applied. Images were acquired on an upright Olympus FV1000 laser-scanning confocal microscope equipped with standard PMTs and highly sensitive GaAsP detectors using the Fluoview 2.1c software. Equivalent stacks of images were compiled for each series and further treated equally for signal output levels using NIH ImageJ (version 1.53o).

Sections were stained with Harris hematoxylin (Diapath) for 3 min, staining intensity was adjusted using a mix of 70% ethanol and 0.3% glacial acetic acid (Amresco, VWR), and another staining step was performed with a 2,000:1 eosin (Diapath):glacial acetic acid solution for 30 s. Samples were then dehydrated, and slides were mounted with coverslips in the xylene-substitute Limonene-Mount mounting medium (Interchim). Images were acquired using a pathology slide scanner (Nanozoomer 2.0HT, Hamamatsu) and extracted using the NDP.view2 software at 20X or 40X magnification.

At least three independent sections were treated by immunohistochemistry and stainings, and representative results are shown in the corresponding figures. For *in vivo* phagocytosis quantification, phagosomes included in the RPE layer were counted in two areas on each side of the optic nerve of sections 1–2 from *n* = 3–4 independent mice (except wt samples at 6.00 AM, for which *n* = 2 mice), the numbers were reported to 100 μm retina width and averaged.

### 2.4 Mononuclear phagocytes quantification on mouse RPE/choroidal and retinal flatmounts

Mice of 8- and 22-months of age were euthanized by CO_2_ asphyxiation and eyes were enucleated (8-month-old *n* = 4, 22-month-old *n* = 3). Globes were fixed in 4% PFA for 45 min, sectioned at the limbus, and the cornea and lens were discarded. RPE/choroid tissues were separated from the retina. RPE/choroids were incubated overnight with a rabbit anti-IBA1 antibody, rat anti-ZO-1, and AlexaFluor647 phalloidin (Supplementary Table S1) in 1X PBS containing 0.1% Triton. Tissues were rinsed and incubated for 2 h with an AlexaFluor488-conjugated donkey anti-rabbit IgG, an AlexaFluor594-conjugated goat anti-rat IgG, and counterstained with Hoechst 33342 in 1X PBS (all 1:1,000, ThermoFisher Scientific). Retinas were incubated overnight with rabbit anti-IBA1 antibody and AlexaFluor 546 peanut agglutinin (PNA) (Supplementary Table S1) in 1X PBS containing 0.1% Triton. Tissues were rinsed and incubated for 2 h with an AlexaFluor 488-conjugated donkey anti-rabbit IgG and counterstained with Hoechst 33342 in 1X PBS (all 1:1,000, ThermoFisher Scientific).

RPE/choroid and retina (photoreceptors side up) tissues were flatmounted, viewed, and photographed with a Leica DM550B fluorescence microscope (Leica Biosystems). MPs were counted on the whole surface of each RPE/choroid and retinal flatmount. Close-up pictures were acquired using an upright Olympus FV1000 confocal microscope coupled with the Fluoview version 2.1c software (Olympus, Rungis, France). Equivalent stacks of images were compiled for each sample using NIH ImageJ (version 1.53o).^1^

### 2.5 Visual phenotyping

Electroretinograms (ERGs) were performed on MerTK^CR^ (*n* = 7–10) and control (*n* = 4–7) mice aged 3–12 months and at 18 months (independent cohorts for each age). After an overnight dark adaptation, mice were anesthetized with ketamine 1,000 (80 mg/kg, Axience) and xylazine (8 mg/kg, Rompun, Bayer HealthCare). Body temperature was maintained at 37°C with a heating pad. Pupils were dilated with tropicamide (Mydriaticum 0.5%, Thea) and phenylephrine chloride (Neosynephrine 5%, CSP). The cornea was locally anesthetized with oxybuprocaïne chlorhydrate (Thea) application. Upper and lower lids were retracted to keep the eye open and proptosed. A small gold wire loop electrode was placed in contact with the cornea to record the retinal response to light stimuli through a layer of Lubrithal (Dechra Veterinary Products) for better contact and to avoid eye dryness. A first needle electrode was placed on the head as a reference, and a second one was placed in the lower back to ground the signal. The light stimulus was provided by a white LED in a Ganzfeld stimulator (ColorDome Lab Cradle, Diagnosys LLC). Scotopic ERG recording was performed using a set of four stimulus intensity levels (0.003, 0.03, 0.3, 3, and 10 cds/m^2^). Responses were averaged over five flash stimulations. Photopic cone ERGs were recorded on a rod-suppressing background light of 20 cd/m^2^, after a 5-min adaption period. A 10 cd/m^2^ stimulus intensity level was used for light-adapted ERGs. Each photopic cone ERG response was averaged over 10 consecutive flashes. Flicker ERGs were recorded at 10 and 20 Hz. Responses were amplified and filtered (1 Hz-low and 300 Hz-high cutoff filters) with a 1-channel DC-/AC-amplifier. A drop of lubricating cream (Lubrithal) was applied on the unrecorded eye during the overall procedure to avoid the development of any corneal opacity, and an application of oxybuprocaine hydrochloride was made on each recorded eye to reduce animal suffering upon waking. Analysis of ERG signals was done using the Espion V6 software.

Optomotor responses (OMRs) to rotating vertical gratings were recorded on the OptoMotry 1.77 system (Cerebral Mechanics, Canada) on the same cohort of animals between the ages of 3 and 12 months to assess the evolution of contrast sensitivity and visual acuity (*n* = 10 MerTK^CR^, 5 males and 5 females, and *n* = 4 male controls). Each mouse was placed unrestrained on a central pedestal facing four computer monitor screens. Once accustomed to this environment, the mouse is presented with black and white striped patterns that rotate either clockwise or counter-clockwise as determined randomly by the OptoMotry software. Mice track the gratings with reflexive head saccades that are recorded by the overhead video camera. Spatial frequency thresholds between 0.03 and 0.5 cycles/degree were measured by systematically increasing the spatial frequency of the grating up to 100% contrast and rotating at a speed of 2 rpm. All analyses were assessed by the same experimenter, who was unaware of the genotype. OMR analysis was performed using the OptoMotry 1.7.7 software. Changes in visual acuity were quantified by OMR as described (Prusky et al., 2004; Douglas et al., 2005).

To study the retinal structure using fundus photography (FP) and optical coherence tomography (OCT), mouse cohorts (*n* = 12 MerTK^CR^ and *n* = 5 controls for the FP/OCT cohort, *n* = 9 MerTK^CR^ and *n* = 5 controls for the OCT only cohort) were followed between the ages of 3 and 12 months and at 18 months. Mice were first anesthetized under isoflurane combined with air at a rate of 5% for induction and 2% for maintenance. Pupils were dilated using tropicamide and phenylephrine chloride eyedrops as described above. For FP and autofluorescence (AF) detection, eyes were coated with a lubricating cream (see above) to promote contact with the lamp and prevent the eyes from drying out. Each mouse was placed on a platform with a breathing mask to maintain the anesthesia. The lens of the lamp was then placed in front of the eye. Two pictures were taken, the first one under white light and the second one using a fluorescence filter (488 nm) to detect any AF. OCT imaging acquisitions were made using several positions of the eye facing the lens to obtain a video recording of the different retinal layers through the entire depth of the eye. Video recordings were then extracted, and images were processed using the InvivoVueClinicDb.db software v1.4.0.4260 (Bioptigen).

### 2.6 RPE and macrophage cell culture

To isolate RPE from 10- to 12-day-old mice for the primary culture, we enucleated eyecups gently to keep perfect contact between RPE and photoreceptors, and successive enzymatic digestion steps were performed as previously described with slight modifications (Nandrot et al., 2004; Farkas et al., 2014). In brief, after removal of the lens, eyes were treated with 1 mg/mL bovine hyaluronidase (Sigma) in Ca^2+^/Mg^2+^-free Hepes-buffered Hanks saline (Gibco) for 45 min at 37°C to allow an easy peeling of the neural retina and to expose the RPE. A second incubation was performed in 1.5 mg/mL trypsin (Difco) in Hepes-buffered Hanks saline for 45 min at 37°C, and patches of RPE were peeled off manually from the Bruch’s membrane and pelleted gently at 1,200 rpm for 2 min. After another short 1.5-min digestion step in 0.25% trypsin–EDTA (Gibco) and centrifugation, purified RPE cells were seeded into serum-coated 384-well plates (Corning). Cells were then grown at 37°C for 4–8 days before further experiments in MEMα medium complemented with 5% FBS (Hyclone, ThermoFisher), 1% N1 supplement, 1% non-essential amino acids (Gibco), 500 μM glutamine (Gibco), 0.25 mg/mL taurine, 0.02 μg/mL hydrocortisone, 0.0130 pg/mL triiodothyronine, and 1% penicillin/streptomycin (Gibco), a medium recipe already published (Maminishkis et al., 2006).

For peritoneal macrophage isolation and culture, 4-month-old mice were euthanized by CO_2_ inhalation, and we followed a previously described protocol (Pineda-Torra et al., 2015). Concisely, mouse abdomens were cleaned with 70% ethanol before incision. Then, the abdominal skin was removed to expose the peritoneum. Into the peritoneal cavity, 5 mL of 1% penicillin/streptomycin 1X PBS was added using a 26G needle, and a little abdomen massage/shaking was done before recovering the liquid. This step was repeated twice, and cells were collected into a 15 mL tube and centrifuged at 300 rpm for 10 min. The pellets were resuspended in RPMI medium (Sigma) supplemented with 1% penicillin/streptomycin, and cells were counted in a Malassez counting chamber. Cells were seeded at a density of 20,000–40,000 cells/well for XFp plates and 120,800 cells/well for 96-well plates (see section 2.10), and the medium was changed after allowing live cells to attach for 1 h. Cells were then grown at 37°C for 24–72 h before metabolic flux and mitochondrial activity measurements.

### 2.7 POS isolation

Retinae were taken out of fresh slaughterhouse porcine eyes, and POS were isolated as previously described (Parinot et al., 2014). In brief, enucleation and tissue collections were performed under dim red light, and retinae were collected in a homogenization solution (20% sucrose, 20 mM tris acetate pH 7.2, 2 mM MgCl_2_, 10 mM glucose, and 5 mM taurine). Retina suspensions were obtained after full shaking and gauze filtering, separated on continuous 25–60% sucrose gradients (in tris acetate pH 7.2, 10 mM glucose, and 5 mM taurine) and ultracentrifuged at 25,000 rpm for 50 min at 4°C (Beckman SW 32 Ti swinging rotor). Isolated POS observed as orange bands were collected, diluted, and washed in a series of three solutions (20 mM tris acetate pH 7.2, 5 mM taurine; 10% sucrose, 20 mM tris acetate pH 7.2, 5 mM taurine; 10% sucrose, 20 mM tris acetate pH 7.2, 5 mM taurine). During these wash steps, POS pellets were obtained by centrifugation at 5,000 rpm at 4°C for 10 min each (Beckman JA25.50 rotor) and finally resuspended in DMEM with 2.5% sucrose, counted, aliquoted, and stored at −80°C.

For labeling, resuspended POS were incubated with 1 mg/mL fluorescein isothiocyanate (FITC) (Molecular Probes) for 2 h at room temperature (RT) on a rotator in 10% sucrose, 20 mM sodium phosphate pH 7.2, and 5 mM taurine. FITC-POS were then washed, counted, and frozen as described above.

### 2.8 POS phagocytosis

For phagocytosis quantification assays, confluent and polarized RPE cells were challenged for 1.5 h at 37°C in 5% CO_2_ with approximately 10 FITC-POS particles per cell in MEMα, as previously described (*n* = 5) (Nandrot et al., 2004; Farkas et al., 2014). Subsequently, to remove excess POS, cells were washed three times with 1X PBS-CM containing 0.2 mM CaCl_2_ and 1 mM MgCl_2_. To quench extracellular fluorescence and determine the quantities of internalized POS, some cells were treated with Trypan blue (Gibco) for 10 min, while non-treated wells permit the measurement of total phagocytosis (Finnemann et al., 1997). Binding is evaluated by the following calculation: total phagocytosis minus internalization. After two washes and a chemical fixation step with 4% PFA for 15 min, all cells were treated with 50 mM NH_4_Cl to quench free aldehyde groups before blocking 1% BSA in PBS-CM. For labeling tight junctions, RPE cells were incubated with an anti-rabbit ZO-1 (Supplementary Table S1) overnight and then washed three times with 1% BSA in PBS-CM for 10 min. Secondary antibodies anti-rabbit AlexaFluor594 (1:250) and 647-conjugated phalloidin (1:40, FluoProbes) were incubated for 1 h, followed by two more washes in 1% BSA in PBS-CM. Nuclei were specifically stained with Hoechst (1:1,000, Invitrogen).

Finally, an automated fluorescence microscope was used to quantify FITC/Hoechst ratios by counting the number of FITC-labeled POS and Hoechst-stained nuclei per cell for each well (Arrayscan VTI, HCS Studio software [spot detector v4.1], Thermo Scientific). Immunocytochemistry images were acquired using a spinning disk confocal microscope (CQ1, Confocal Quantitative Image Cytometer, CellpathFinder Software, Yokogawa) with a 20X zoom.

### 2.9 Electron microscopy

After careful dissection out of the eye socket, complete eyeballs from 18-month-old mice were immersed in the fixing solution (1.5% glutaraldehyde, 1% PFA in 0.1 M cacodylate buffer, pH 7.4) for 2 h at room temperature on a rotator (*n* = 5). For each sample, the cornea and ora serrata were removed, and the eyecup opened in petals. Four different 1-mm^3^ RPE/choroid pieces were dissected out and further fixed at 4°C until processing by the electron microscopy core facility (Institut de Biologie Paris-Seine, Sorbonne Université, Paris, France). After three 15-min washing steps in a 0.1 M cacodylate buffer, a post-fixation step was proceeded with in a 0.1 M cacodylate buffer containing 1% OsO_4_ for 1 to 2 h on ice. Samples were then washed five times for 15 min in ddH_2_O. Afterward, tissue pieces were dehydrated using gradual ethanol bath concentrations (50, 70, 95, and 100%) for 15 min each, followed by two 15-min baths in 100% anhydrous acetone. On a rotator, samples were successively impregnated with different acetone:resin ratios and incubation times (1 h at 3:1 ratio, overnight at 1:1, and 1 h at 1:3), and then with pure resin (two solution changes during the day, followed by an overnight bath). Finally, a polymerization step was carried out at 60°C for 48 h. Ultrathin cuts with a length between 500 μm and 1 mm were obtained using an ultramicrotome (UCT, Leica). Pictures were acquired on a JEM-2100HC scanning transmission electron microscope (JEOL) at 6,000–25,000X.

### 2.10 Metabolic flux and mitochondrial activity analysis

For Seahorse (Agilent) metabolic flux analysis, RPE/choroid and retina samples from 3-month-old mice were separated, and 1-mm RPE/choroid punches (three for two eyes) were immediately collected in individual wells of a FluxPak Seahorse XFp cartridge in the Seahorse XF base medium minimal DMEM without phenol red (Agilent Technologies) already supplemented with 1 mM pyruvate, 2 mM glutamine, and 10 mM glucose, adjusted to pH7.4 (*n* = 1 of each genotype in triplicates per experimental plate, with *n* = 4–7 experiments for tissues samples and *n* = 3–5 for macrophages). Punches were equilibrated for 1 h at 37°C in an incubator without CO_2_. We used mitochondrial inhibitors oligomycin (1.5 μM), FCCP (carbonyl cyanide 4-(trifluoromethoxy)-phenylhydrazone, 0.5 μM), and rotenone/antimycin A (0.5 μM) as detailed in the XFp Cell Mito Stress Test Kit (Agilent Technologies). Oxygen consumption rate (OCR) and ExtraCellular Acidification Rate (ECAR) were automatically calculated and recorded on the machine by the Seahorse Report Generator online software (Agilent Technologies). Modifications to the original program were implemented to meet our specific experiment requirements: each step is separated into four intervals between drug addition, and three measurements are performed for each step in cycles of 3 min mixing/1 min waiting/2 min measuring. The XFp Glycolytic Rate Assay Kit (Agilent Technologies) was used according to the manufacturer’s instructions.

To evaluate the mitochondrial activity, RPE/choroid fractions (*n* = 4) and peritoneal macrophages primary cultures (*n* = 6) from wild-type and MerTK^CR^ mice were incubated in a black 96-well microplate (Corning) for 30 min at 37°C with MitoTracker Red FM and MitoTracker Green FM fluorescent probes (Invitrogen). MitoTracker Red FM stains active mitochondria, whereas MitoTracker Green FM stains total mitochondria. Both fluorescent probes are diluted in the Seahorse XF base medium minimal DMEM without phenol red (Agilent Technologies) supplemented with 1 mM pyruvate, 2 mM glutamine, and 10 mM glucose, and adjusted to pH7.4 to reach a 200-nM concentration. After two washes with this medium, a microplate reader (Spark, Tecan) counts the different fluorescent spots with 0 μs of lag time and 40 μs of integration time. The TR-F mode is used at 37°C (MitoTracker Red FM 581 ± 10 nm excitation and 640 ± 10 nm emission filters; Mitotracker Green FM 490 ± 10 nm excitation and 516 ± 10 nm emission filters). The percentage of active mitochondria on total mitochondria is then calculated.

After all assays, protein levels were determined for each sample to normalize the results using the Bradford method. A second normalization step was performed for each XFp plate measured on the Seahorse against the average starting value measured for wild-type samples. Experiments were repeated 5–7 times for each genotype, averaged, and the most distant outliers were removed.

### 2.11 Statistical analysis

Statistical significance of results was determined using the unpaired *t*-test with Welch’s correction or the Holm-Sidak method for multiple comparisons or with 2-way ANOVA with a Sidak correction for multiple comparisons, all providing adjusted *p*-values, each row being analyzed individually without assuming equal s.d. Significance thresholds were set as follows: *p < 0.05, **p < 0.01, ***p < 0.001, ****p < 0.0001.

## 3 Results

### 3.1 Normal structure and visual function of MerTK^CR^ retinae

**Figure 1:**
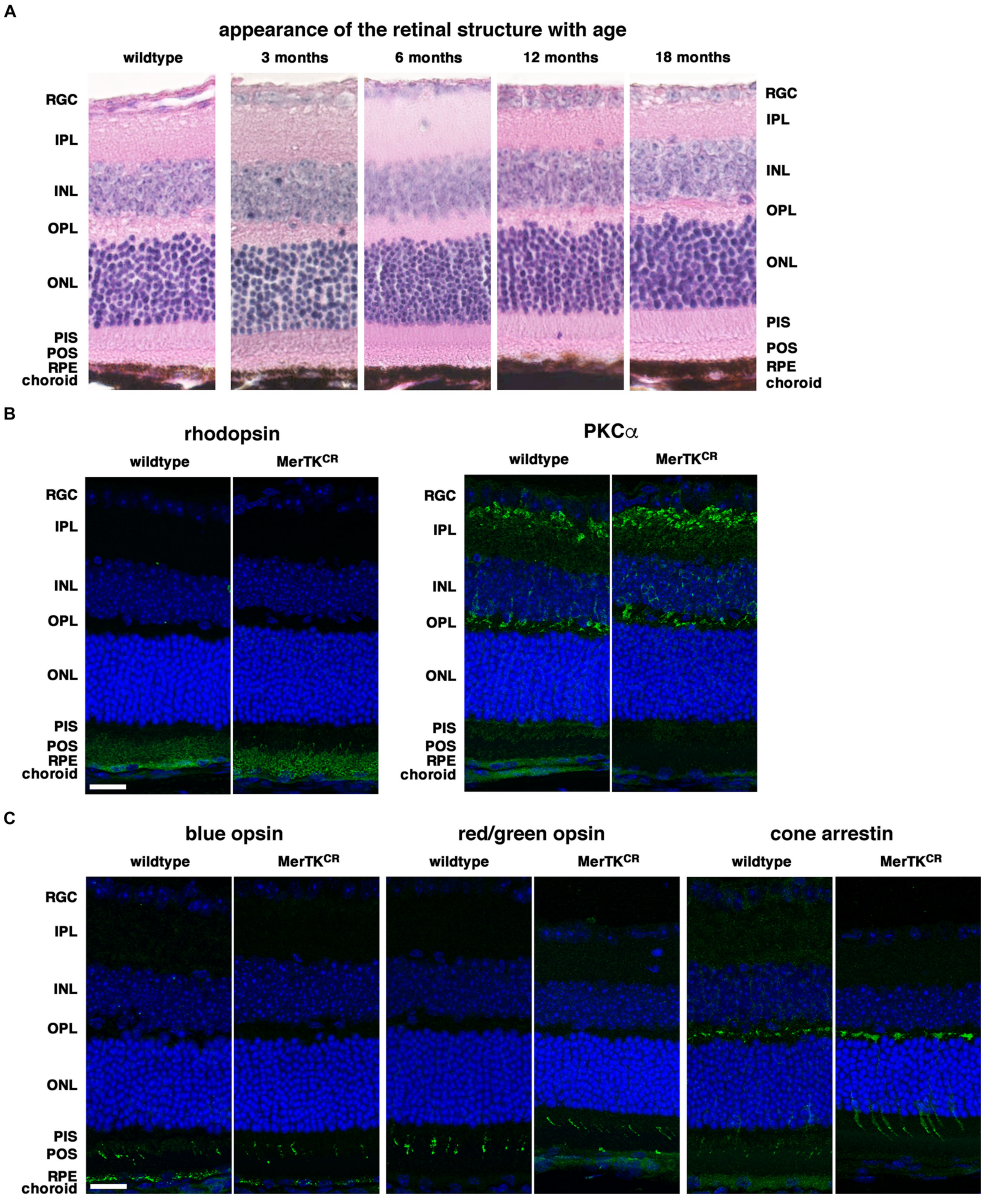
Normal retinal structure and organization in MerTK^CR^ mice. **(A)** Representative hematoxylin/eosin stainings show that MerTK^CR^ retinae between the ages of 3 and 18 months (panels with ages) display a normal structure similar to 12-month-old wild-type controls, as indicated. **(B)** Representative immunofluorescence stainings of rhodopsin and PKCα show that rod photoreceptors are organized normally in MerTK^CR^ mice when compared to wild-type controls, as indicated. **(C)** Representative immunofluorescence stainings of blue and red/green opsins and of cone arrestin show that cone photoreceptor structure is normal in MerTK^CR^ mice when compared to wild-type controls, as indicated. RGC, retinal ganglion cells; IPL, inner plexiform layer; INL, inner nuclear layer; OPL, outer plexiform layer; ONL, outer nuclear layer; PIS, photoreceptor inner segments; POS, photoreceptor outer segments; RPE, retinal pigment epithelium. Markers are in green, and nuclei in blue. Scale bars: 20 μm.

We first analyzed the overall structure and organization of both retina and RPE in the absence of MerTK activity downregulation. Hematoxylin/eosin stainings clearly show that the MerTK^CR^ retinae developed normally and did not appear to change with age up to 18 months (Figure 1A). Photoreceptor inner and outer segments were well-aligned facing RPE cells. Rhodopsin, PKCα, blue and cone opsins, and cone arrestin labelings demonstrated that MerTK^CR^ rods and cones organization is normal (Figures 1B,C).

**Figure 2:**
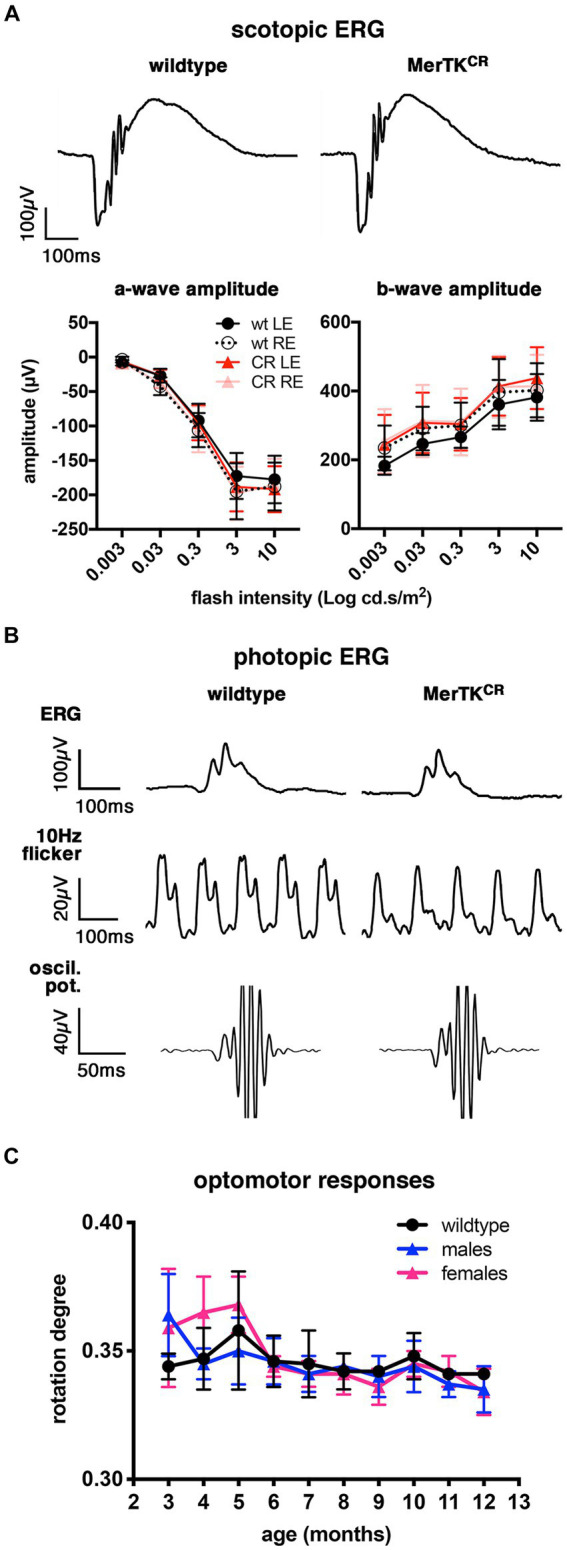
Normal ERG and optomotor responses in MerTK^CR^ mice. **(A)** Representative single scotopic ERG responses (top) of 18-month-old wild-type and MerTK^CR^ mice and cohort quantifications (bottom) for a- (left) and b-wave (right) amplitudes at different flash intensities as indicated. wt, wild-type (black); CR, MerTK^CR^ (red); LE, left eyes (plain lines); RE, right eyes (dotted or clearer lines). Mean ± s.d., *n* = 5–8, non-significant. **(B)** Representative photopic ERG, 10 Hz flicker, and oscillating potentials (oscil. pot.) responses of 18-month-old wild-type and MerTK^CR^ mice as indicated. **(C)** Optomotor responses of 3- to 12-month-old wild-type (black) and male (blue) and female (pink) MerTK^CR^ mice as indicated. Mean ± s.d., *n* = 4–5, non-significant.

To evaluate the visual capacities of MerTK^CR^ mice at the eye and brain levels, we followed up electroretinograms (ERG) and optomotor (OMR) responses in independent cohorts between the ages of 3 and 12 months and at 18 months. Scotopic profiles and measures of a- and b-wave amplitudes (Figure 2A), as well as implicit times (data not shown), were similar between wild-type controls and transgenic MerTK^CR^ mice. Photopic ERG and oscillating potentials were also equivalent in both strains, and only flickers seemed to be slightly attenuated in MerTK^CR^ mice (Figure 2B). Optomotor responses did not differ either, and slightly decreased with age in all animals, whatever their genotype (Figure 2C).

### 3.2 Light sensitivity phenotype with macrophage infiltration in MerTK^CR^ retinae

**Figure 3:**
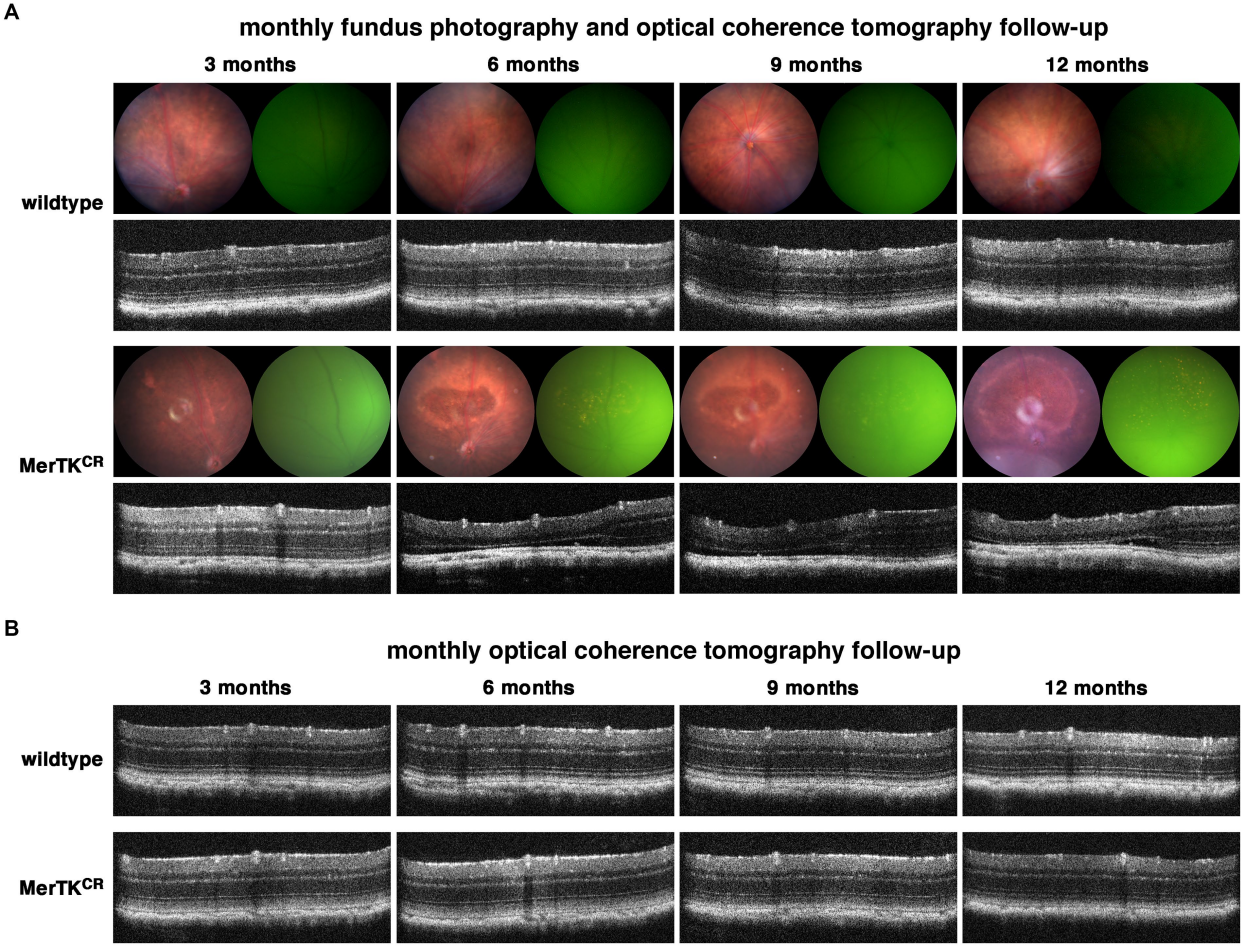
Light sensitivity phenotype in MerTK^CR^ retinae. **(A,B)** The monthly follow-up between the ages of 3 and 12 months with FP and OCT **(A)** led to the appearance of degenerated areas in MerTK^CR^ but not with a monthly follow-up with OCT alone **(B)**.

To characterize the evolution of each mouse eye fundus with age, we imaged a separate cohort of mice using monthly fundus photography (FP) and optical coherence tomography (OCT) between the ages of 1 and 12 months. In MerTK^CR^ mice, we observed the appearance of degenerative areas in the dorsal retina close to the optic nerve, with a marked rim and in the middle of which RPE cells became visible (Figure 3A). OCT thickness was reduced in these areas mostly at the outer nuclear layer level, thus confirming photoreceptor cells loss. Interestingly, these areas also displayed autofluorescence AF punctae at the fundus, which seemed to correspond to “bumps” observed between RPE cells and dying photoreceptors on OCT images. Once detected, these degenerative areas did not seem to spread too much in size with time. Indeed, we repeated monthly FP on naive 10-month-old animals, and MerTK^CR^ mice did develop degenerative areas of similar size and location as younger mice (Supplementary Figure S1A). As another noticeable feature of these degenerative areas, we noticed that the phenotype did not develop at the same pace in all mice. Indeed, males seemed to be affected earlier than females, i.e., with fewer FP repeats, and left eyes showed degeneration before right eyes (Supplementary Figure S1B).

Strikingly, fundus photographs of mice from the ERG and OMR cohorts taken just before sacrifice suggest that no abnormalities were detectable in any control or transgenic mouse. To understand this discrepancy, and after excluding differences in lighting and food regimen, we set out another cohort that we followed only with monthly OCTs to test if the phenotype was due to the repeated fundus photographies. We did not observe any degeneration in these mice, thus suggesting that the phenotype might indeed be related to repeated illumination of their retina with the fundus lamp (Figure 3B).

**Figure 4:**
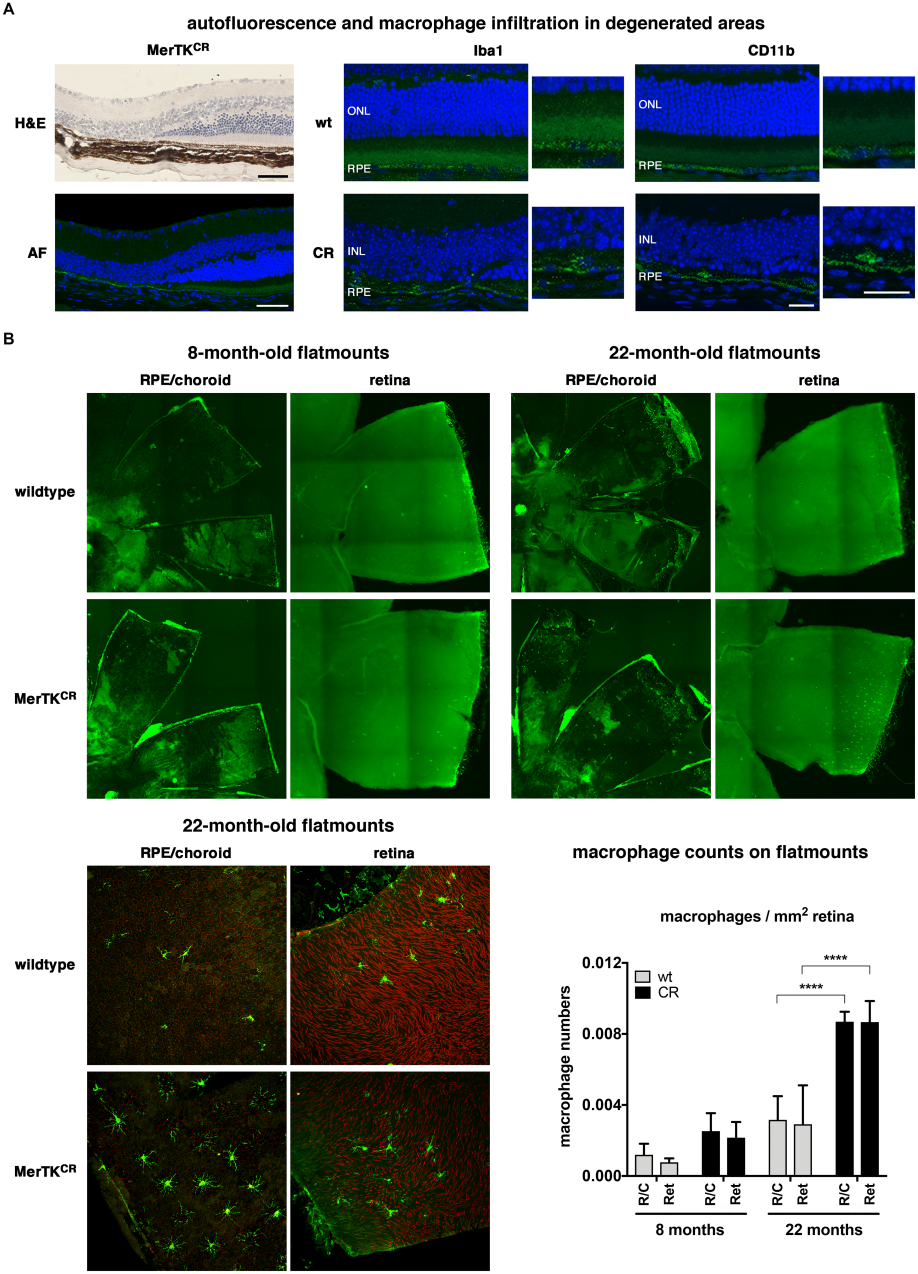
Increased presence of macrophages in MerTK^CR^ retinae. **(A)** Degenerated areas observed on MerTK^CR^ hematoxylin/eosin stained sections (H&E, top left picture) were autofluorescent (AF, green, bottom left picture). Iba1 (green, middle panels) and CD11b (green, right panels) macroglia/macrophage markers labeled cells inserted between RPE cells (RPE) and degenerating photoreceptors (ONL) in MerTK^CR^ (CR) retinal sections but not in wild-type controls (wt), as indicated. The nuclei of bipolar, horizontal, and amacrine cells (INL) were seen in areas where photoreceptors had degenerated. Nuclei labeled with DAPI are in blue. Scale bars: 50 μm (H&E, AF), 20 μm (Iba1, CD11b). **(B)** RPE/choroid and retina flatmounts from 8-month-old (top left panels) and 22-month-old (top right panels) mice show a larger number of Iba1-labeled cells (green) in MerTK^CR^ tissues than in wild-type controls, as indicated. Close-up confocal pictures on 22-month-old samples (bottom left) confirm the increased presence of Iba1-labeled cells (green) in MerTK^CR^ tissues than in wild-type controls, as indicated. RPE cell tight junctions labeled using ZO-1 (RPE/choroid, red) and cone photoreceptors using peanut agglutinin (retina, red). Quantification (bottom right) corroborates that greater numbers of Iba1-labeled cells are present in RPE/choroid and retinal samples from MerTK^CR^ mice (CR, black bars) when compared to wild-type controls (wt, gray bars), as indicated. Mean ± s.d., *n* = 3–4, **** *p* < 0.0001.

We analyzed more closely the areas of photoreceptor loss on sections stained with hematoxylin and eosin or after simple nuclei labeling and confocal microscope imaging (Figure 4A). We could clearly see the loss of photoreceptor nuclei that led to the overall thinning of the retina. In contrast, other retinal layers did not seem impacted. Interestingly, degenerated areas showed increased AF inside RPE cells detected on retinal sections. To further characterize the “bumps” observed on OCT scans, we labeled sections using the microglia/macrophage-specific Iba1 and CD11b markers, recognizing monocytes and macrophages. Both proteins were expressed in RPE cells, especially in degenerated areas. Moreover, we detected a signal around the nuclei of cells inserted between RPE cells and degenerated photoreceptors in MerTK^CR^ retinae. To verify that the presence of microglial cells/macrophages was increased in MerTK^CR^ mice, we performed RPE/choroid and retina flatmounts at 8 and 22 months of age (Figure 4B). The Iba1 labeling confirms that, in both young and old mice, more microglial cells/macrophages were detected between the RPE and photoreceptors in MerTK^CR^ mice when compared to wild-type mice, a difference that was significant at 22 months of age.

### 3.3 Deregulated POS phagocytosis by MerTK^CR^ RPE

**Figure 5:**
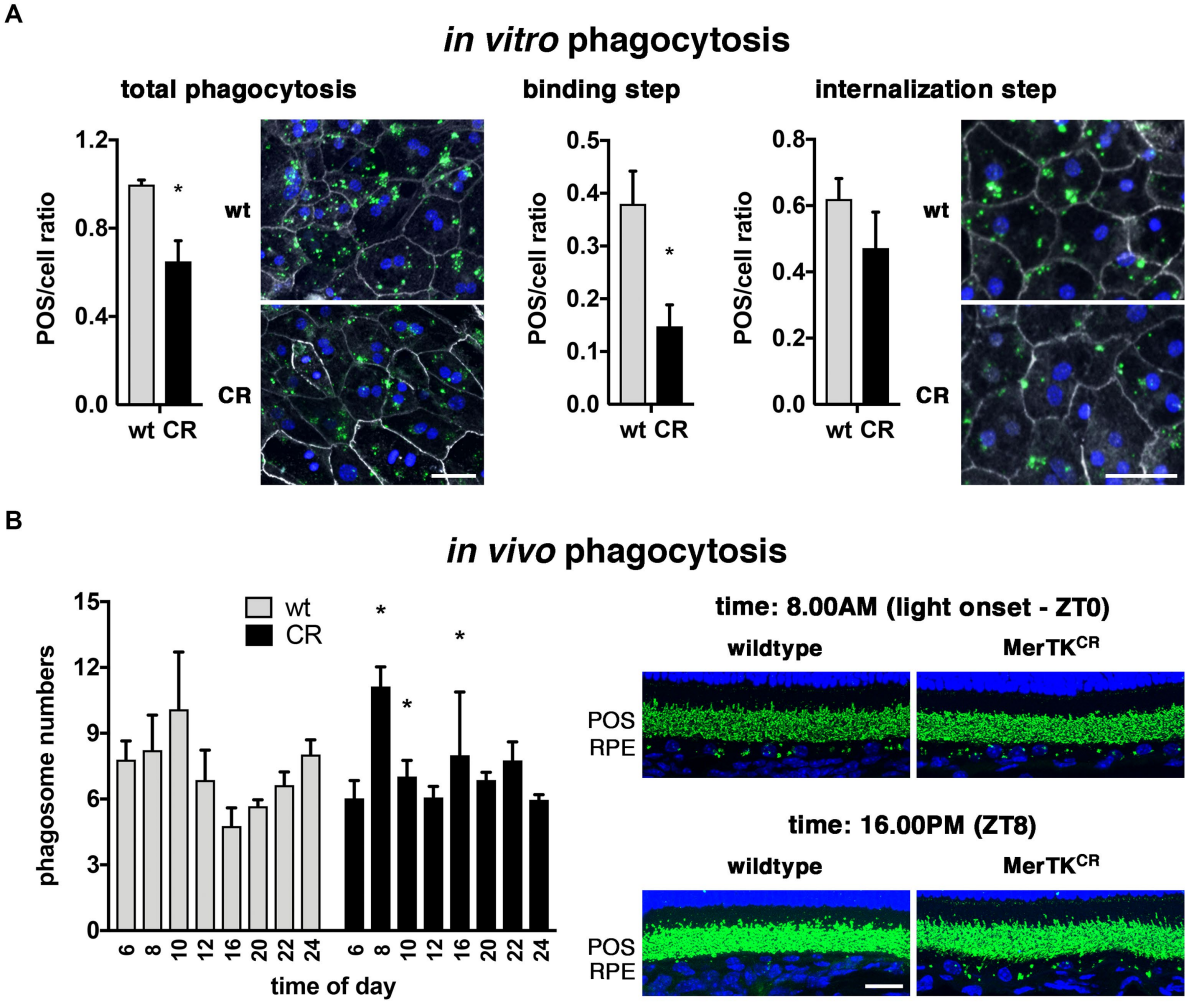
Decreased *in vitro* and deregulated *in vivo* phagocytosis in MerTK^CR^ mice. **(A)** Total *in vitro* phagocytosis (left) was significantly decreased in MerTK^CR^ (CR, black bars, and bottom pictures) RPE when compared to control cells (wt, gray bars, and top pictures). The binding step of phagocytosis (middle) seemed more diminished than the internalization step (right). FITC-POS: green; cell junctions (ZO-1): gray; nuclei: blue. **(B)** The *in vivo* rhythm of phagocytosis was significantly deregulated in MerTK^CR^ (CR, black bars, and right-hand pictures) RPE when compared to control cells (wt, gray bars, and left-hand pictures) at 8.00 AM (light onset, ZT0), 10.00 AM (expected phagocytic peak), and 16.00 PM (ZT8), as indicated. Rhodopsin: green; nuclei: blue. Scale bars: 50 μm **(A)** and 20 μm **(B)**. Mean ± s.d., *n* = 5 **(A)** and *n* = 3–4 (**(B)**, except controls at 6.00 AM *n* = 2), *p < 0.05.

POS phagocytosis is a multi-step process initiated by the specific recognition of POS (binding) to alphavbeta5 integrin receptors and the subsequent activation of intracellular signaling pathways leading to MerTK activation (Nandrot et al., 2004). MerTK function is required for POS uptake inside the cells, the internalization step of phagocytosis (Feng et al., 2002). As we showed previously, MerTK cleavage appears to regulate its activity, so we assessed the impact of the absence of MerTK cleavage and, thus, the deregulation of MerTK function on RPE phagocytosis (Law et al., 2015). Total phagocytosis, corresponding to the number of POS bound at the cell surface plus the number of POS that have been internalized, of mouse primary RPE cells from MerTK^CR^ mice was decreased by 35% (± 11%) when compared to wild-type control cells (Figure 5A). When looking more closely at the two subsequent steps of phagocytosis, the reduction was more pronounced for POS binding (−23% ± 14%) than for their internalization (−15% ± 8%, non-significant). However, the internalization percentage compared to total phagocytosis was similar for control (62% ± 14%) and MerTK^CR^ (72% ± 37%) RPE cell cultures.

One of the cardinal features of POS phagocytosis by RPE cells is the circadian regulation of its activity (LaVail, 1976; Nandrot et al., 2004). We thus proceeded to characterize the daily rhythm of POS phagocytosis in MerTK^CR^ mice along eight time-points of the light:dark cycle. The overall profile was modified with a peak of activity occurring at light onset (8.00 AM) earlier than in wild-type mice, followed by an immediate significant decrease at the time of the normal phagocytic peak (10.00 AM) (Figure 5B). Interestingly, in the afternoon phagocytosis is usually at its lowest, but in MerTK^CR^ mice we detected a significant increase of phagocytosis at 16.00 PM. In addition, in these two marked peaks, the overall MerTK^CR^ profile appears more homogenous during the rest of the light:dark cycle than in control animals. Notably, the total amount of phagocytosis over the quantified 24-h period was equivalent in both strains, amounting to 58.9 ± 1.7 phagosomes per 100 μm retina for MerTK^CR^ mice versus 58.1 ± 1.7 phagosomes for control mice. Similarly, levels of total MerTK proteins were equal in both strain RPE/choroid and retinal samples (data not shown).

### 3.4 Defective mitochondria in MerTK^CR^ RPE but not macrophages

**Figure 6:**
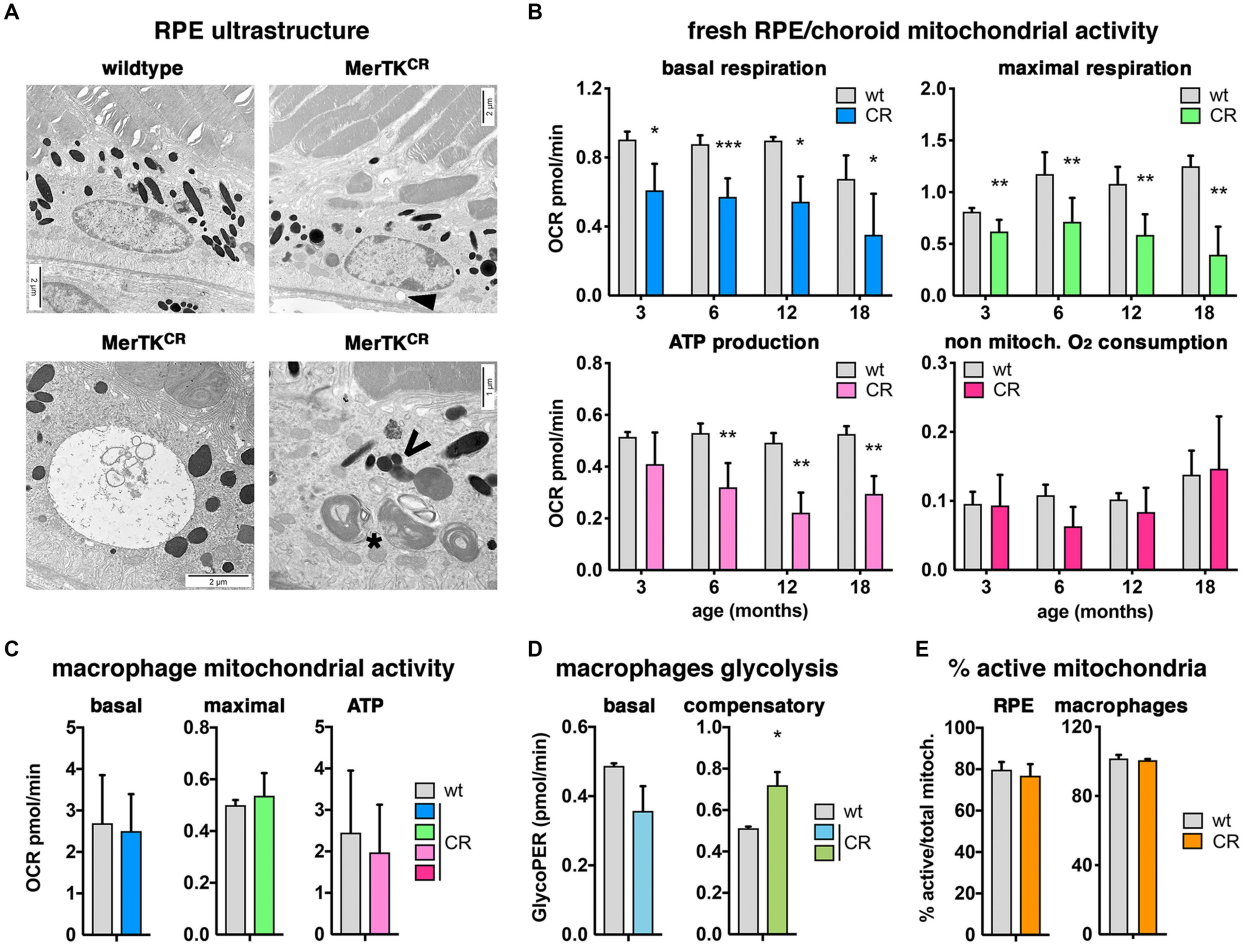
Impaired mitochondrial function in MerTK^CR^ RPE but not macrophages. **(A)** Representative electron microscopy pictures showing ultrastructural changes in MerTK^CR^ RPE such as vacuoles (top right [arrowhead] and bottom left pictures) and abnormal phagolysosomes (bottom right picture) in comparison with wild-type control RPE (top left picture). *, phagosome including POS disks; V, defective phagolysosome. Scale bars: 2 μm. **(B,C)** Basal (blue) and maximal (green) respiration, as well as ATP production (light pink) but not the non-mitochondrial respiration (dark pink), were decreased in MerTK^CR^ (CR, colored bars) RPE (B, *n* = 4–7) but not in MerTK^CR^ peritoneal macrophages (**(C)**, *n* = 3–5) when compared to wild-type control samples (wt, gray bars). **(D)** Basal (light blue) and compensatory (darker green) glycolysis seemed deregulated in MerTK^CR^ but not in wild-type peritoneal macrophages (*n* = 6). **(E)** The percentage of active mitochondria was similar in MerTK^CR^ (CR, orange bars) and control (wt, gray bars) RPE (left panel, *n* = 4) as well as in MerTK^CR^ and control peritoneal macrophages (right panel, *n* = 6). Mean ± s.d., *p < 0.05, **p < 0.01, ***p < 0.001.

We investigated in more detail the structure and function of MerTK^CR^ RPE cells. At the ultrastructural level, RPE basal infoldings and apical microvilli seemed normal in MerTK^CR^ mice (Figure 6A). Similarly, POS appeared well organized and aligned, and with proper contact with RPE microvilli, and phagosomes were present. However, vacuoles of different sizes were visible in older MerTK^CR^ but not wild-type control mice. Moreover, electron-dense phagolysosomes were present in transgenic mice, suggesting some defective POS processing.

To understand if the observed differences in the phagocytic function and potential cellular stress could be linked to defective metabolism, we then investigated the energetic metabolism of freshly dissected RPE/choroid samples (Figure 6B). We detected a significant decrease in both basal and maximal respiration as well as of ATP production at 3 months that seems to worsen with age. In these samples, no difference was seen in the non-mitochondrial oxygen consumption, thus suggesting that other energy sources might not be affected. As they also express MerTK, we investigated macrophages from the peritoneum, and, in contrast with RPE/choroid samples, they did not show any difference in any factor studied (Figure 6C). However, the analysis of macrophage glycolysis identified a lower basal and significant higher compensatory glycolytic function in mutant peritoneal macrophages (Figure 6D). Interestingly, both MerTK^CR^ RPE/choroid and macrophages had similar percentages of active mitochondria when compared to wild-type controls (Figure 6E).

## 4 Discussion

MerTK and other receptors of the Tyro3/Axl/MerTK (TAM) family have long been related to phagocytosis defects in various tissues and processes (Nandrot et al., 2000; Feng et al., 2002; Hafizi and Dahlbäck, 2006b). Recently, the cleavage of the extracellular portion of MerTK (sMerTK) has been shown to contribute to the regulation of its function by acting as a decoy receptor (Sather et al., 2007; Nandrot et al., 2012; Law et al., 2015). The MerTK^CR^ model is devoid of the cleavage site and thus prevents the production of decoy sMerTK receptors. However, in contrast to what we expected from previous studies by us and others on macrophages, we were surprised to observe that phagocytosis was decreased instead of being enhanced (Law et al., 2015; Cai et al., 2016). Interestingly, the rate of internalization was not impacted, suggesting that this effect might be related to the regulatory role of MerTK on POS binding and not to its direct implication in POS internalization. Indeed, previous studies suggested that MerTK is part of a negative feedback loop controlling the amounts of POS that can be tethered by alphavbeta5 integrin receptors (Wu et al., 2005; Nandrot et al., 2012). Hence, it is entirely possible that overactive MerTK receptors accentuate this process, leading to lesser numbers of POS attached to the RPE surface and thus phagocytosed. This discrepancy might also be linked to more general features, such as the difference between *in vitro* and *in vivo* conditions for RPE cells, as encountering POS upon challenge is different from being permanently in contact with them. Alternatively, other regulatory mechanisms might intervene, such as different half-lives or transcriptional rates between the *in vitro* and the *in vivo* contexts or between wild-type and MerTK^CR^ tissues.

*In vivo*, the circadian profile of POS elimination is deregulated, with the phagocytic peak occurring earlier at light onset and a supplementary peak mid-afternoon when phagocytosis is usually at its lowest. These data are consistent with overactive MerTK receptors that might anticipate the phagocytic peak and generate an extra peak in the afternoon. Interestingly, we previously showed that MerTK cleavage seemed to be somewhat cyclic, with a small increase at light onset and at peak phagocytosis time, but the highest levels were detected between 16.00 PM and 20.00 PM (Law et al., 2015). Hence, the absence of cleavage in the afternoon time could explain the extra peak at 16.00 PM that extends up to 22.00 PM detected in our model. It is not the first time an *in vitro* phagocytic defect is translated into a modified profile of *in vivo* phagocytosis. Indeed, in alphavbeta5 integrin knockout mice displaying a 70% decrease of POS uptake by RPE cells in culture, *in vivo* phagocytosis is present at similar levels over the full 24-h period when compared to wild-type mice but totally arrhythmic (Nandrot et al., 2004). This could be explained by the fact that, *in vitro*, phagocytosis starts upon RPE cells’ challenge with POS, while *in vivo*, the contact is permanent, and phagocytosis is launched under a circadian fashion by alphavbeta5 integrin receptors that then stimulate POS internalization by the activation of MerTK receptors via intracellular signaling pathways (Nandrot et al., 2004). Interestingly, however, decreased phagocytosis after 1.5 h of POS challenge *in vitro* is consistent with the decreased phagocytosis observed *in vivo* at the time of the phagocytic peak that occurs 1.5–2 h after light onset. In parallel, MerTK^CR^ RPE cells seem to accumulate phagolysosomes near the apical surface, as seen on electron microscopy micrographs, suggestive of a potentially defective or retarded POS digestion. It is interesting to notice that our work confirms recent data showing that C57Bl6J background mice display a more attenuated circadian profile than 129SvJEms background mice, with a peak less marked and spanning over a slightly larger timeframe (Nandrot et al., 2004; Farkas et al., 2014; Vargas and Finnemann, 2022).

For each RPE cell, this daily phagocytic function involves eliminating approximately 7–10% of at least 25–30 PRs per day, which requires high amounts of energy. We thus investigated mitochondrial metabolism and identified that basal and maximal respiration profiles, as well as ATP production, are defective in MerTK^CR^ RPE/choroid fractions at all ages studied. Notably, these defects present in young animals increase with age but are totally absent in peritoneal macrophages, in which phagocytic function is increased (Cai et al., 2017), showing a tissue-specific global mitochondrial dysfunction that is not linked to lower percentages of active mitochondria (Finnemann and Rodriguez-Boulan, 1999). However, when looking more closely at glycolysis, MerTK^CR^ peritoneal macrophages seem to display a slight deregulation of this energy source. While we did not test the mitochondrial activity earlier than at 3 months of age, we expect these defects to be present early on. As the number of active mitochondria is similar between controls and transgenic mice, most probably their function is directly affected. However, at this stage, it is not clear if there is a link between deregulated phagocytosis and changes in mitochondrial respiration. Lipids from POS could indeed be used as an energy source; however, in our model, other energy sources outside of mitochondria do not seem to be intervening in this defect in RPE cells, as non-mitochondrial O_2_ consumption is similar in mutant and control mice. At the electron microscopy level, signs of accumulating cellular stress are visible in RPE cells, such as vacuoles and unresolved phagolysosomes.

Recently, an RNAseq study on *Mertk^−/−^* mice identified changes in the expression of 60 genes involved in several functions, including phagocytosis and metabolism, before retinal degeneration occurs (Penberthy et al., 2017). Hence it appears that there might be a direct link between the absence or the deregulation of MerTK receptors and metabolic pathways, besides its well-known function in phagocytosis. In addition, phagocytosis has been shown to stimulate the local production of molecules such as insulin that might influence overall retinal homeostasis related to glycolysis and maybe other metabolic aspects in and around RPE cells (Etchegaray et al., 2023). On the other hand, Tyro3, another member of the TAM tyrosine kinase receptors family, has been shown to function as a modifier allele for MerTK (Vollrath et al., 2015; Mercau et al., 2023). It is thus possible that some other factors influence MerTK function outside of its cleavage that might explain some of the discrepancies we observed between *in vitro* and *in vivo* results. Furthermore, while RPE cells express both MerTK and Tyro3, macrophages only express MerTK (Zagórska et al., 2014). This could explain why we observe different phenotypes in these two cell types and might explain the tissue-specificity of their regulation and local functions.

The retinal structure and function appear normal in MerTK^CR^ mice, as shown by histology, electroretinography, and optomotor tests. Retinal layers are properly organized at all ages tested, and rods, cones, and related secondary neurons are normally spanned along the retina. Both scotopic and photopic ERG responses are similar to control mice, except maybe a slightly lower sensitivity to 10 Hz flicker flashes when both rods and cones are responsive (Wu and Burns, 1996). Interestingly, flicker lights are also associated with the rapid dilation of retinal vessels (Chou et al., 2019), suggesting that the more limited responses observed with the flicker ERG in MerTK^CR^ mice might be due to more limited dilation of the vessels, as rods and cones normally respond to other ERG stimuli. Optomotor responses along different ages are undistinguishable from wild-type control ones, showing that the light signals are transmitted properly to the visual areas in the brain. However, when following the retinal fundus appearance on a monthly basis using fundus photography and OCT, we noticed the appearance of degenerative areas always occurring in the same location in the eye. Notably, males were affected earlier than females, suggesting that the sex of each animal may influence pathogenesis, a phenomenon already described in other types of retinal degeneration models (Li et al., 2019). Surprisingly, this degeneration does not occur when only OCT is used monthly, suggesting a potential increased sensitivity to light exposure in MerTK^CR^ mice.

The lesions we observed appear to be geographic, an intriguing characteristic. The setting of the illumination can not explain this peculiar lesion phenotype, as the illumination is neither geographic nor dorsal. Interestingly, in the regions of PRs loss, infiltration of microglial cells/macrophages is observed, showing inflammatory processes in the area. These macrophages are later resolved, which raised one hypothesis: could these lesions be created by overactive macrophages that are attracted by multiple repetitions of light exposure (“stress”) during the fundus photography follow-up period? In our experiments, we could not visualize these microglial cells/macrophages before the appearance of the degenerative areas. However, we showed that more microglia/macrophages are present in peripheral retinal tissues from MerTK^CR^ mice at two different ages, which could render the retina more reactive to repeated stress. These microglial/macrophagic cells might be recruited in the lesioned area and contribute to the degenerative phenotype. Noteworthily, both loss (RCS rats) and gain of MerTK function lead to inflammation in the retina, highlighting the importance of proper MerTK functioning to keep the retinal homeostasis in an equilibrated state (Lew et al., 2020). Macrophages have previously been shown to invade the retina in several pathological processes, including those due to increased resistance of microglial cells/macrophages or the loss of the immunosuppressive function of RPE cells (Sennlaub et al., 2013; Mathis et al., 2017; Beguier et al., 2020). Inflammation has been increasingly implicated in retinal degenerative processes, including pathologies related to MerTK receptors (Mercau et al., 2023). In addition, inhibition of microglial cells has been associated with a delay in the development of MerTK-associated retinal degeneration (Lew et al., 2020). On the other hand, MerTK^CR^ mice display decreased inflammation in other tissues, diminished accumulation of atherosclerotic plaques, and increased circulation of pro-resolving lipid mediators (Cai et al., 2016, 2017). Moreover, MerTK signaling promotes the expression of inflammation resolution mediators (Cai et al., 2018). In the retina, MerTK is activated directly by intracellular pathways downstream of alphavbeta5 integrin receptors at the time of peak phagocytosis (Nandrot et al., 2004). Hence, it will be interesting to see the exact contribution of MerTK cleavage versus intracellular pathways to control MerTK activity and its implication in the maintenance of low levels of inflammation, a characteristic which might be different in RPE cells and macrophages as MerTK ligands seem to act differently in these two cell types (Law et al., 2015).

Taken together, our results give us new insights into the regulation of MerTK function and its various functions in the retina. In this model, both microglial cells/macrophages and RPE cells express MerTK, and their respective activities might be affected by the absence of MerTK cleavage. Further experiments will allow us to identify the origin of macrophage infiltration to discriminate between two hypotheses, the overactivity of macrophages, and the decreased immunosuppressive role of RPE cells. The origin of the light sensitivity observed is currently under investigation to identify which cell type underlies this characteristic. Taken together, our data highlight a potential new role for MerTK in the maintenance of the immune privilege in the retina and suggest MerTK could also be involved in other pathological pathways aside from phagocytosis defects, potentially in relation with the energetic metabolism.

## Data availability statement

The raw data supporting the conclusions of this article will be made available by the authors, without undue reservation.

## Ethics statement

The animal study was approved by the Charles Darwin Animal Experimentation Ethics Committee from Sorbonne Université; the French Ministry for Education, Higher Studies and Research. The study was conducted in accordance with the local legislation and institutional requirements.

## Author contributions

JE: Data curation, Formal analysis, Investigation, Writing – original draft. QR: Data curation, Formal analysis, Investigation, Writing – original draft, Supervision, Writing – review & editing. SRé: Data curation, Formal analysis, Investigation, Writing – original draft. EV: Data curation, Formal analysis, Investigation, Writing – original draft. SRo: Data curation, Formal analysis, Investigation, Writing – review & editing. JD: Data curation, Formal analysis, Writing – original draft. QC: Data curation, Formal analysis, Writing – original draft. SA: Data curation, Methodology, Resources, Writing – original draft, Writing – review & editing. CN: Data curation, Resources, Writing – review & editing. BC: Resources, Writing – review & editing. VF: Methodology, Writing – review & editing. FS: Methodology, Resources, Writing – review & editing. EN: Resources, Writing – review & editing, Conceptualization, Data curation, Formal analysis, Funding acquisition, Investigation, Methodology, Project administration, Supervision, Validation, Visualization, Writing – original draft.

## Footnotes

^1^ https://imagej.nih.gov/ij/
