# Supplementary material for: Retinal atrophy, inflammation, phagocytic and metabolic disruptions develop in the MerTK-cleavage-resistant mouse model
Source: Front Neurosci. 2024 Apr 12;18:1256522. doi: 10.3389/fnins.2024.1256522 (PMC11047123; doi:10.3389/fnins.2024.1256522)
Supplement: Supplementary file 1 [file Table_1.docx]

**Supplementary Table 1.** Name, reference, species and dilutions corresponding to the various antibodies used (mAb = monoclonal antibody).

| ***Antibody*** | ***Reference*** | ***Species*** | ***IHC dilution*** |
| --- | --- | --- | --- |
| CD11b | Bio-Rad MCA711 | Rat mAb | 1:200 |
| Cone arrestin | Chemicon AB15282 | Rabbit | 1:200 |
| Iba1 | Fujifilm Wako 019-19741 | Rabbit | 1:500 |
| Opsin (blue) | Chemicon AB5407 | Rabbit | 1:500 |
| Opsin (red/green) | Chemicon AB5405 | Rabbit | 1:500 |
| Peanut Agglutinin – AlexaFluor594 | ThermoFisher Scientific L32459 | / | 1:100 |
| Phalloidin – AlexaFluor647 | ThermoFisher Scientific A30107 | / | 1:100 |
| Protein Kinase Cα | Sigma P4334 | Rabbit | 1:1,000 |
| Rhodopsin [RET-P1] | Chemicon MAB5316 | Mouse mAb | 1:400 |
| ZO-1 [R40.76] (flatmounts) | Merck Millipore MABT11 | Rat | 1:200 |
| ZO-1 (primary cultures) | Invitrogen 61-7300 | Rabbit | 1:250 |
