# Supplementary figures and images for: Retinal atrophy, inflammation, phagocytic and metabolic disruptions develop in the MerTK-cleavage-resistant mouse model
Source: Front Neurosci. 2024 Apr 12;18:1256522. doi: 10.3389/fnins.2024.1256522 (PMC11047123; doi:10.3389/fnins.2024.1256522)

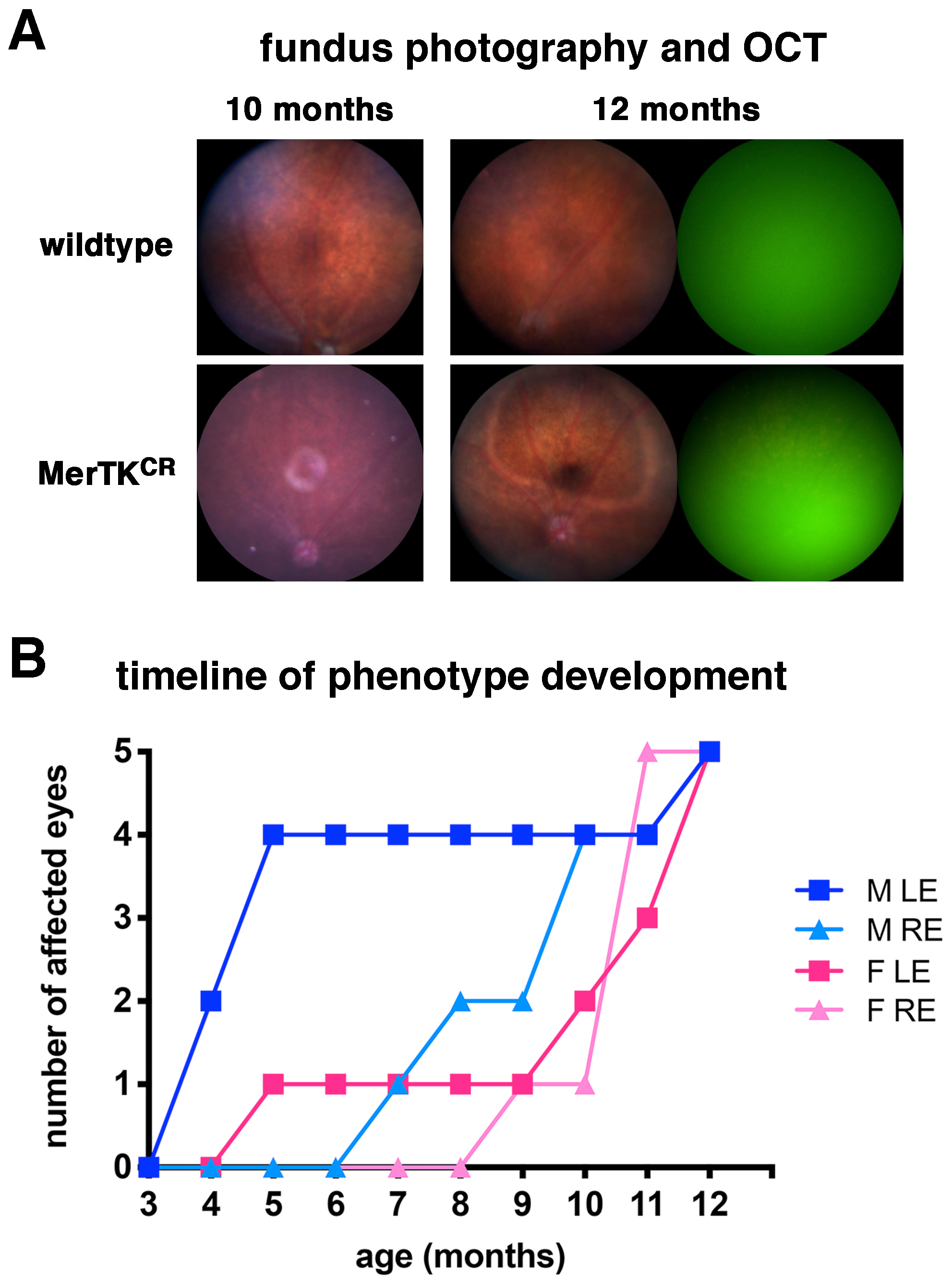

Supplement: Supplementary file 2 [file Image_1.TIF]
